# The molecular epidemiology, genotyping, and clinical manifestation of prevalent adenovirus infection during the epidemic keratoconjunctivitis, South of Iran

**DOI:** 10.1186/s40001-022-00928-0

**Published:** 2023-03-02

**Authors:** Vahidreza Afrasiabi, Rozita Ghojoghi, Seyed Younes Hosseini, Jamal Sarvari, Fatemeh Nekooei, Negar Joharinia, Sahar Hadian, Mohammad Gholami, Mahmood Nejabat

**Affiliations:** 1 Poostchi Ophtalmalogy Research Center, Department of Ophtalmology, School of Medicine, Shiraz University of Medical Sciences, Shiraz, Iran; 2 Department of Bacteriology & Virology, School of Medicine, Shiraz University of Medical Sciences, Shiraz, Iran; 3 Gastroenterohepatology Research Center, Shiraz University of Medical Sciences, Shiraz, Iran

**Keywords:** Adenovirus, Genotyping, Clinical finding, Epidemic Keratoconjunctivitis

## Abstract

**Purpose:**

Adenoviral-mediated keratoconjunctivitis is among the emergency diseases of ophthalmology with long-term sequels. The role of adenovirus infection, ocular-related genotypes, and association with ocular symptoms need to be investigated for epidemiological as well as clinical purposes.

**Methods:**

The affected patients from two close keratoconjunctivitis epidemics were included in the study. The swab samples were taken from patients; the total DNA was extracted and then used as a template for in-house Real-time PCR. Besides, partial *Hexon* genes of 11 adenovirus positive samples were amplified and submitted to sanger sequencing. Moreover, they were finally evaluated by phylogenetic analysis.

**Results:**

Of 153 patients, 92 (60.1%) were males and 47 cases (30.7%) had a history of eye infection in the family or colleagues. Real-time PCR tests of 126 samples (82.4%) were positive for adenovirus, and all eleven cases that underwent sequencing analysis were determined to be group 8 (HAdV-D8). Adenovirus infection has a significant relationship with infection among family or colleagues (*p* = 0.048), membrane formation (*p* = 0.047), conjunctival bleeding (*p* = 0.046), tearing, and pain(*p* < 0.05).

**Conclusions:**

The results indicated that Adenovirus is the major cause of keratoconjunctivitis, and HAdV-D8 was the most common genotype in the area. There were some clinical manifestations associated with Adenovirus infection of the conjunctiva.

## Introduction

Adenovirus is the leading cause of Epidemic Keratoconjunctivitis (EKC) worldwide. Conjunctivitis accounts for a large number of visits to ophthalmology clinics and imposes a heavy financial burden on the health system. Accordingly, 1% of all doctor visits in the United States are related to conjunctivitis [1]. Most cases of conjunctivitis are viral-based, and discrimination from a bacterial basis is of great importance to reduce antibiotic consumption and better clinical management. In addition, 65–90% of all viral conjunctivitis cases are caused by adenovirus, which makes it the most important agent in this regard [2]. Uncovering the role of Adenovirus in keratoconjunctivitis from other viral agents for example *Coscackiviruses* and *Herpes simplex virus* could be beneficial as some specific antivirals are under development [38].

Adenovirus is a naked double-stranded DNA virus with a special projection, named fiber [2]. Human adenoviruses are divided into 7 groups from A to G and 68 subgroups based on the biological properties [3]. Among all groups, members of group D, including genotype 8, 19, and 37, are the major causes of ECK, followed by other genotypes, such as 2, 4, 7, 11, 14, 16, and 29 [3, 4].

Adenoviral conjunctivitis is diagnosed by cell culture, Polymerase chain reaction (PCR), enzyme immunoassays, direct immunofluorescence tests, immune dot-blot tests, and point of care tests [5]. Real-time PCR is now known as the “gold standard” method for diagnosing viral conjunctivitis. Studies suggest that Real-time PCR is more sensitive for detecting adenoviruses than other virological methods [6]. Adenovirus serotyping can be simply performed by surface antigen sequencing. Identification of the adenovirus genotypes which are involved in EKC is required to understand the geographical distribution and draw a relationship between a specific genotype and clinical features [7]. Identification of the exact types of adenovirus associated with EKC might contribute to a better understanding of the severity of diseases like conjunctivitis [36]; however, the correlation between Adenovirus infection and the severity or clinical findings are poorly discernable from other causes in practice.

A study at Wills Ophthalmology Hospital in Philadelphia found that the prevalence of adenoviral conjunctivitis was 62% [8]. In a large study in Turkey, adenoviral DNA was detected by PCR in 213 our of 488 samples (43%), collected from patients with viral conjunctivitis. The genotypic analysis revealed that seven genotypes, including 3, 4, 8, 11, 19, 37, and 53, were circulating during the epidemic episode, while genotypes 4 and 8 were the dominant genotypes found in 66.3 and 24.7% of the samples, respectively [7]. In addition, the prevalence of EKC does not appear to be related to gender, race, social status, or nutritional status [9].

In the Middle East, studies regarding adenovirus prevalence and genotyping during keratoconjunctivitis are limited to a few molecular epidemiology studies that indicate the dominant prevalence of adenovirus genotype HAd8 [10], 11. Determination of virus genotype in epidemics allows utilizing more effective preventive protocols in society.

Viral conjunctivitis is highly contagious and is transmitted through infected personal items, direct contact with infected fingers, eye secretions, medical equipment, and swimming pools. In addition, tears, saliva, respiratory droplets, and feces are involved in transmission [12]. Other ways through which EKC can spread are tonometry, slit-lamp exams, putting in contact lenses, and contact with infected doctors [13].

Although adenoviral conjunctivitis is commonly a self-limiting disease with no long-term complications, in severe cases of EKC, significant chronic complications can occur that affect life. Severe EKC can lead to secondary dry eye, fibrosis of the glands and lacrimal ducts, corneal scarring, development of irregular astigmatism, and even permanent vision loss. Conjunctival scarring can occur in patients with severe disease after the formation of a membrane [14]. The impact of adenovirus infection on the severity of ocular disease and its correlation with viral genotype is a matter of doubt. Differential diagnosis of each EKG-related infection is hard to achieve, but finding the specific clues for each agent is practical. The traumatic-inflammatory extent of the involved conjunctiva and following the sequels of adenovirus infection could be differentiated from others.

Owing to the complications of EKC, the present study aimed to determine the frequency of adenovirus infection, circulating genotype, and correlation of infection with symptoms during two close epidemics, in a reference clinical center, Shiraz, the largest city in the south of Iran in 2019.

## Materials and methods

### Patients and clinical evaluation

In this cross-sectional and descriptive study, using an easy sampling method, we enrolled 165 eligible patients referred to the ophthalmology emergency department of referral Khalili Teaching Hospital in the study during 2 close epidemics in the summer and autumn seasons of 2019. The confirmed bacterial conjunctivitis cases were excluded from the study. Also, the patients with long-term use of eye drops, glaucoma, history of herpes simplex keratitis, eye surgery in the past week, use of contact lenses in the past few days, and history of allergic conjunctivitis were excluded.

After obtaining informed consent, the patients' information was collected in the form of a questionnaire provided by the examiner physician. The study was approved by the Ethics Committee of Shiraz University of Medical Sciences (IR.SUMS.MED.REC.1399.507).

Guideline statement: A research has been carried out in accordance with relevant guidelines and regulations.

Any patient with conjunctivitis whose symptoms had started two weeks ago was asked to be included in the study. Their clinical pictures were evaluated by an ophthalmologist based on the history and ophthalmological examinations. Inclusion criterion was referring to the hospital with symptoms of conjunctivitis, such as redness, pain, tearing, follicular reaction, eye discharge, and preauricular lymphadenopathy. Also, exclusion criteria were history of long-term use of eye drops, glaucoma history of herpes simplex keratitis, eye surgery in the past week, use of contact lenses in the past few days—and history of allergic conjunctivitis.

### Sampling

The samples were collected from the lower palpebral conjunctiva using a sterile Dacron swab with a plastic shaft, which was subsequently introduced into a transport medium (DMEM culture media, PH = 7.3) supplemented with Pen−Strep antibiotics and amphotericin B antifungal. Specimens were transported on ice to the lab and stored at − 70 °C until used.

### Real-time PCR development

An in-house real-time PCR assay targeting a small part of the *Hexon* gene was developed for the detection of the adenoviral genome. Real-time PCR was performed in a SYBR-Green-based method using a pair of primers to detect all human-related adenoviral genotypes (Table 1). Real-time PCR was performed using the RealQ plus master mix Green (Ampliqon Inc.) mixture containing 5 μL template, 1X master mix, heat-labile uracil-N-glycosylase, and 300 nM of each specific Adenovirus primer. The reaction was done using the ABI7500 instrument under the thermal cycling condition of an initial denaturation step at 95 °C for 10 min, followed by 40 amplification cycles of denaturation at 95 °C for 20 s, annealing and elongation at 60 °C for 15 s. At the end of thermal cycling, melting temperature (Tm) analysis was also applied in order to avoid false positive interpretations by primer dimers and nonspecific bands.

**Table 1:** The primer list employed in the study

Primer pair	Primer sequences	Length of product
Hexon detection	Forward:5'-TGCAACATGACMAARGACTGG-3' Reserve: 5'- RCTCATRGGCTGGAAGTT-3'	132 bp
Hexon sequencing	Forward: 5'- GACAACCGGGTGCTAGACATG-3' Reserve: 5'-GTAGTTGGGTCTGTTGGGGCAT-3'	690 bp

So as to the test validity and specificity, four negative controls, including Staphylococcus aureus extracted genome, human *herpes simplex virus-1* (HSV-1) extracted genome, a plasmid expressing eGFP (Q.Biogene Inc.), as well as total DNA extracted from human cells were also included. Besides, Human Adenovirus serotype 36, a recombinant plasmid containing adenovirus backbone type 5 (Q.biogene Inc.), and a clinical serotype 8 (that had been confirmed by sequencing, HAdD8) were also included as positive controls, to investigate the specificity.

The detection limit of the method was investigated on a diluted standard sample of the recombinant plasmid containing an Adenovirus type 5 genome backbone. The concentration of plasmid was measured using Nanodrop™ absorbance reading and the conversion of mass to copy number was done by online converter tools. A serial dilution of up to 10^–9^ was prepared and introduced into the experiment to find out the lowest copy number at which the developed test works well.

### Molecular phylogenetic analyses and serotyping

The total DNA was extracted from the samples using the DNP Nucleic Acid Isolation Kit (CinnaGen Inc., Tehran, Iran) according to the manufacturer’s instructions. The extracted DNA was stored at − 70 °C.

In order to detect the prevalent genotypes, were amplified a fragment of the *hexon* gene using a specific Ad-Hex primer set (Table 1). The primer pairs were designed using the NCBI primer designing tool and evaluated on positive clinical samples as well as available positive controls. The thermal cycling conditions were as follows: initial denaturation at 95 °C for 5 min, followed by 35 cycles: 30 s at 95 °C, 30 s at 55 °C, and another 30 s at 72 °C. In this study, 12 samples from different time points of epidemics were randomly selected to be sent for sequencing. PCR products were purified using the MN gel purification kit (Macherey–Nagel Inc.) before being sent for Sanger sequencing.

All the sequences were retrieved from the files and screened by BLAST online tool to find the similarity with the relative sequence. Next, they underwent multiple sequence alignments with numerous reference adenovirus sequences from different groups by Mega10 software. The confirmed reference/clinical strains included human adenoviruses group A, B, C, D, E, and F. Afterward, a phylogenetic tree was constructed by Clustal-X using the distance-based method, Neighbor-Joining, and then validated following 1000 bootstrap replicates.

### Statistical analysis

The data were statistically defined in terms of median and range or mean and standard deviation (± SD), and frequencies. For comparing the categorical data, the Chi-square test and Fisher’s exact test were done. *p *value < 0.05 was considered statistically significant. All statistical calculations were done using SPSS version 22 (SPSS Inc., Chicago, IL, USA).

## Results

### Demographic data and history

Out of the 153 patients included in the study, 92 (60.1%) were males and 61 (39.3%) females. Participants had a mean age of 32.8 years and an age range of 0.5 to 72 years. Based on the history of contact with the infected person or possibly environmental causes, the results showed that 47 patients (30.7%) had a history of eye infection in family members or colleagues during the last 2 weeks before the referring time, and 106 patients (69.3%) had no history of contact with the patients or possible contaminated environment.

Out of the 153 patients, 51 (33.3%) had an infection in the left eye, 54 (35.3%) in the right eye, and 48 (31.4%) in both eyes. Most of the patients were in the age group of 30 to 39 years old (44, 28.8%) and 1 patient (0.77%) was over 70 years old.

### Analytical specificity and detection limit of the real-time PCR

In silico analysis of primers by the primer BLAST tool revealed a highly specific pattern for the recognition of all human adenovirus serotypes. On the other hand, no sign of possible OFF-target for the human genome or other viral sequences, especially *Herpesvirus* and *Enterovirus,* has been detected. The in silico application of the detection primer pair on multiple sequences alignment also showed their suitable strength and specificity for a wide range of adenovirus types. The recent in-house real-time PCR method was determined to be able to detect all serotypes classified in serogroups A to F. The test showed high specificity when it was tested on several irrelevant samples, while it could detect positive DNA samples. The detection limit of the test is estimated to be 50–100 copies/ml as compared to the serially diluted plasmid containing an adenovirus type 5 backbone.

### Molecular detection by real-time PCR

The results of real-time PCR showed that 126 samples (82.4%) were positive for adenovirus, and 27 samples (17.6%) were negative. Investigation of the relationship between demographic characteristics of the studied patients and the real-time PCR results showed that there was a significant relationship between a positive test in terms of conjunctivitis and gender (*P* = 0.023) and a history of infection in the family or colleagues (*P* = 0.048), but this relationship in terms of involved eyes (*P* = 0.637) was not significant. Also, the most positive cases of conjunctivitis were in the age group of 30 to 39 (26.2%), and there was no significant relationship between age and the infection rate (*P* = 0.224). Only one patient was over 70 years old.

### Clinical manifestations

On clinical examination, patients were assessed based on the frequency of Punctate Epithelial Erosions (PEE), corneal epithelial defects (CED), subepithelial infiltration (SEI), Punctate Epithelial Keratitis (PEK), conjunctival injection (CI), chemosis, discharge, conjunctival hemorrhage, existence of membranes, follicular reaction, eyelid inflammation, eyelid redness, photophobia, blurred vision, foreign body sensation (FBS), tearing, itching, pain, cold symptoms, and lymphadenopathy (LAP). The most common clinical findings in patients were conjunctival injection (90.8%), eye discharge (86.6%), conjunctival hemorrhage (85%), tearing (80.4%), follicular reaction (75.8%), and foreign body sensation (67.3%), respectively. The detailed clinical findings are summarized in Table 2.

**Table 2:** The clinical findings in history and physical exam related to cornea, conjunctiva, and eyelid

Patients’ history		Cornea		conjunctiva and eyelid	
Clinical finding	Frequency (%)	Clinical finding	Frequency (%)	Clinical finding	Frequency No (%)
Tearing	123(**80.4%)**	PEE	39(25.5%)	CI	139(**90.8%)**
FBS	103(**67.3%)**	PEK	12(7.8%)	Follicular reaction	116(**75.8%)**
Itching	83(**54.2%)**	CED	8(5.2%)	Petechia	107(**69.9%)**
Cold symptoms	51(33.3%)	SEI	7(4.6%)	Purulent eye Discharge	79(**51.6%)**
Blurred vision	47(30.7%)	–	–	Eyelid swelling	76(49.7%)
Photophobia	37(24.2%)	–	–	Chemosis	63(41.2%)
pain	35(22.9%)	–	–	Nonpurulent eye discharge	53(34.6%)
–	–	–	–	Red eyelids	41(26.8%)
–	–	–	–	LAP	38(24.8%)
–	–	-	–	Pseudo membrane	32(20.9%)
–	–	–	–	Sub Conjunctival hemorrhage	23(15%)
–	–	–	–	True membrane	8(5.2%)

The bold values highlighted to show the significant difference between frequency

*FBS* Foreign Body Sensation, *PEE* Punctate Epithelial Erosions, *PEK* Punctate Epithelial Keratitis, *CED* Corneal Epithelial Defects, *SEI* Subepithelial Infiltration, *CI* Conjunctival Injection, *LAP* Lymphadenopathy

Also, there was a relationship between some clinical findings and a positive Adenovirus genome test. Overall, the results revealed that positive PCR results had a significant relationship with the history of infection in family or colleagues (*p* = 0.048), gender, pain, tearing, membrane formation (*p* = 0.047), conjunctiva bleeding (*p* = 0.046), and pain as demonstrated in Tables 3, 4, and 5. Moreover, the sensation of a foreign thing was significant (*p* = 0.51).

**Table 3:** Relationship between corneal clinical findings and Adenovirus genome detection

Clinical finding	Results	Real-time PCR			
		Positive	Negative	Total	*P* value *
Punctate Epithelial Erosions (PEE)	Yes	31	8	39	**0.58**
	No	95	19	114	
Corneal epithelial defects (CED)	Yes	7	1	8	**>0.5**
	No	119	26	145	
Subepithelial infiltration (SEI)	Yes	6	1	7	**>0.5**
	No	120	26	146	
Punctate Epithelial Keratitis (PEK)	Yes	10	2	12	**>0.05**
	No	116	25	141	

The bold values highlighted to show the significant difference between frequency

**Table 4:** Relationship between conjunctiva and eyelid clinical manifestation and real-time PCR results

Clinical finding			Results		Real-time PCR			
					Positive	Negative	Total	*P* value*
Conjunctival Injection (CI)			Yes		115	24	139	0.71
			No		11	3	14	
Chemosis			Yes		52	11	63	0.96
			No		74	16	90	
Follicular reaction			Yes		96	21	116	0.86
			No		30	6	36	
Eyelid inflammation			Yes		62	14	76	0.80
			No		64	13	77	
Eyelid redness			Yes		36	5	41	0.28
			No		90	22	112	
Lymphadenopathy (LAP)			Yes		36	2	38	0.25
			No		90	25	115	
Discharge			Yes	Purulent	65	14	79	0.97
				Nonpurulent	44	9	53	
			No		17	4	21	
**Conjunctival bleeding**		Yes	Petechiae		90	17	107	**0.046**
			Sub. Conj. Hemorrhage		21	2	23	
			No		15	8	23	
**Membrane**	Yes		True		8	0	8	**0.047**
			Pseudo		30	2	32	
			No		88	25	113	

The underlined bold values highlighted to show the statistical significance of the clinical findings

**Table 5:** The relationship between clinical findings and real-time PCR results

Clinical finding	Results	Real-time PCR			
		Positive	Negative	Total	*P* value *
Photophobia	Yes	34	3	37	0.088
	No	91	24	115	
Blurred vision	Yes	41	6	47	0.28
	No	84	21	105	
**Foreign body sensation (FBS)**	Yes	89	14	103	**0.051**
	No	36	13	49	
Tearing	Yes	105	18	123	**0.048**
	No	21	9	30	
Itching	Yes	68	15	83	0.91
	No	57	12	69	
**Pain**	Yes	33	2	35	**0.042**
	No	92	25	117	
Cold symptoms	Yes	41	10	51	0.65
	No	85	17	102	

The underlined bold values highlighted to show the statistical significance of the clinical findings

### Phylogenetic analysis and tree

**Fig. 1:**
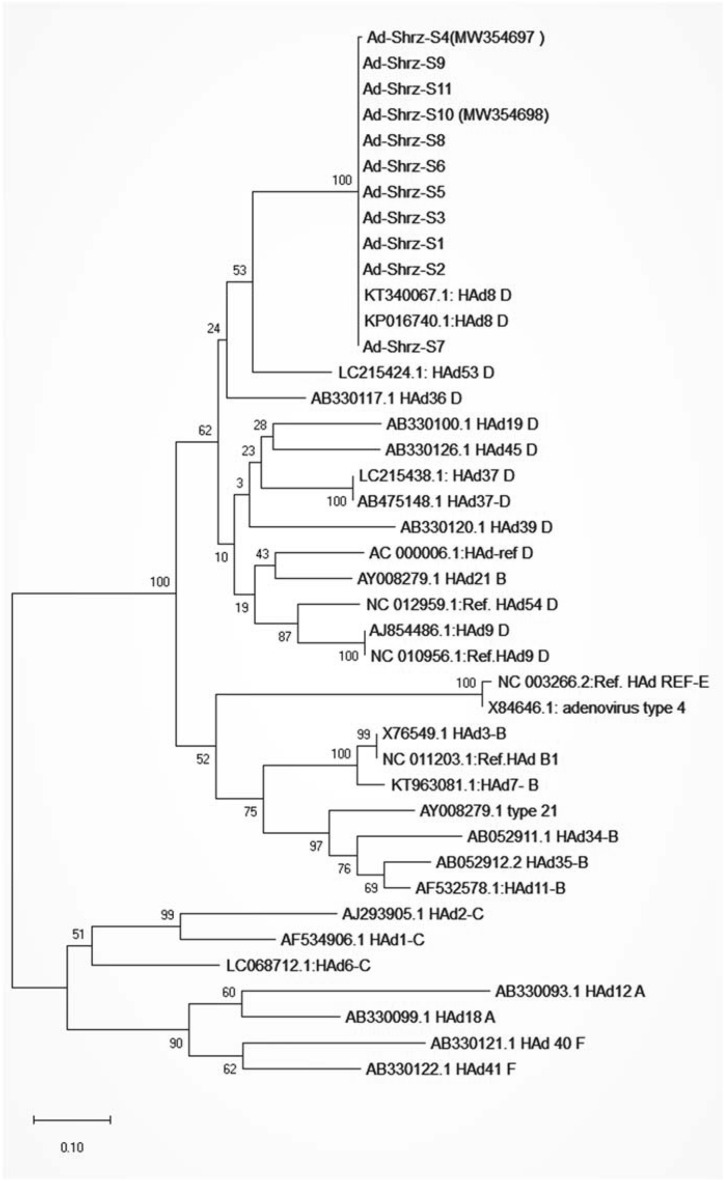
The phylogenetic analysis of the investigated sequences. The sequence comparison of clinical samples with confirmed clinical sequence from NCBI database has shown that all 11 sequenced samples (The top branch: From Ad-Shrz-S4 to Ad-Shrz-S7) were categorized in the Adenovirus group D cluster and at the HAd8 genotype cluster

The fragment encompassing approximately 700 bp of the Hexon gene was amplified to determine the genotype of the viruses. The screening of sequences in BLAST deeply showed the similarity of all the sequences to adenovirus type 8. In addition, the phylogenetic analysis by Neighbor-Joining showed that all 12 sequenced samples were placed in the same cluster of adenovirus type 8, which were nearly identical together with no clear distance on branches, as shown in Fig. 1. As the sequences were too similar to each other, owing to the outbreak, selected sample sequences were deposited in GenBank under the access numbers MW354697 (Ad-Shrz-S4) and MW354698 (Ad-Shrz-S10).

The pattern of the phylogenetic tree also showed a suitable differentiation among the investigated reference sequences, which was a marker of tree reliability created by this part of the Hexon gene.

## Discussion

Outbreaks of adenoviral keratoconjunctivitis are a serious public health concern in ophthalmology departments [15]. Most cases of conjunctivitis are viral-based, and their discrimination from a bacterial infection is of great value due to the reduction in antibiotic use and emergence of new antivirals for specific control of viral causes. Nearly, 65–90% of all viral conjunctivitis cases are caused by adenovirus that highlights the importance of its diagnosis from other viral agents [38]. In addition, adenovirus-mediated conjunctivitis is a highly contagious infection with a transmission rate of up to 50% [16].

There are multiple adenovirus genotypes/serotypes which are associated with different types and severity of infection [12]. Group D, including types 8, 19, 37, and 11 (in group B), are the predominant viruses of keratoconjunctivitis outbreaks from different regions worldwide [17, 18]; however, the number of studies conducted on the prevalent serotypes in the Middle-East is limited. In our region, some studies were conducted to determine the prevalence of adenoviruses in patients with acute conjunctivitis [19]. Also, Shafiei et al. in a similar study demonstrated adenovirus serotype 8 as the most common cause of conjunctivitis in Ahvaz, southwest of Iran [11]. In this regard, the current study aimed to determine the prevalence of adenovirus and related serotypes during two close long-period EKC at Khalili referral Hospital, in the biggest city in the south of the country.

**Fig. 2:**
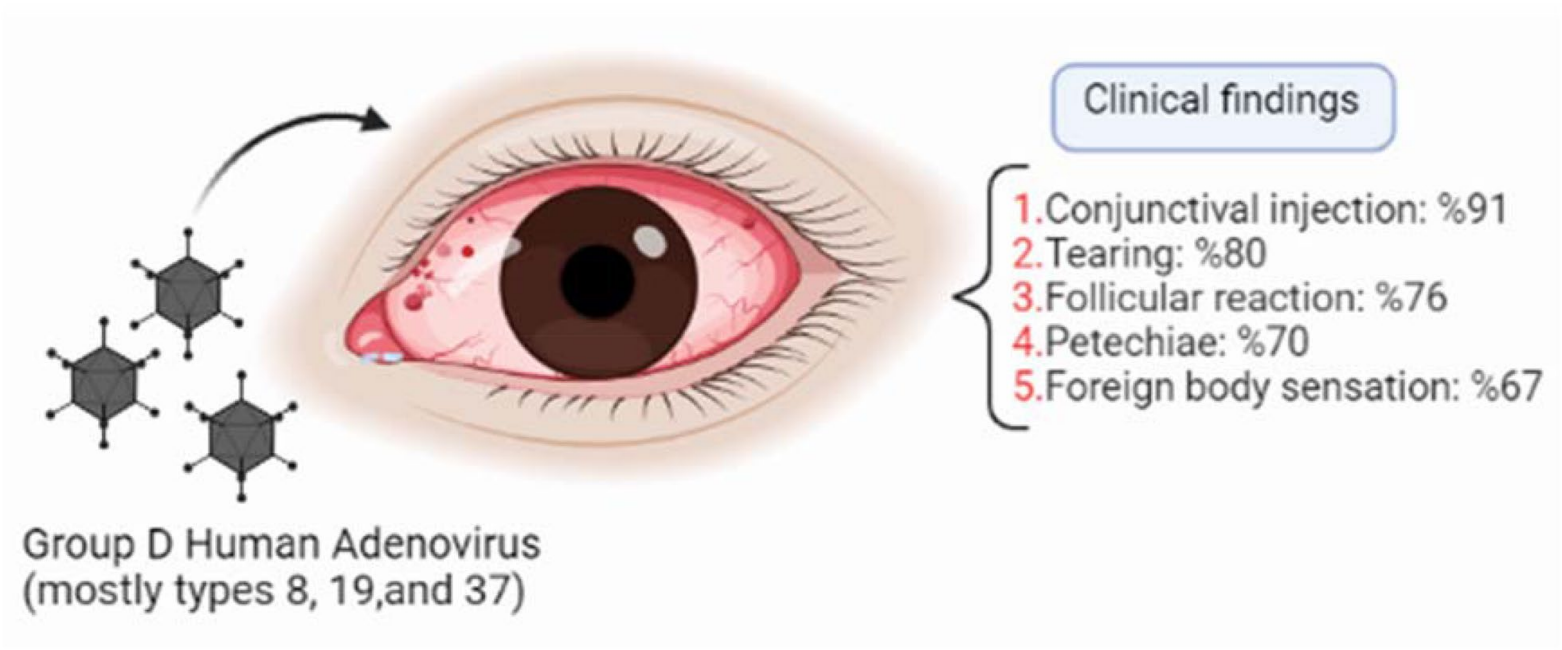
The diagram of Adenovirus eye infection and common clinical findings

In the recent study, most of 153 patients were males, and real-time PCR indicated that 82.4% of samples were adenovirus positive. The age group of 30–39 years old was the most affected population, while a rare case of > 65 years old was diagnosed. In a study in Iraq, most of the subjects were female and most of the HAds affected age was 30–49 y/o group (34%), and the age group over 50 was the least vulnerable population to severe disease. In a review analysis, it was indicated that all age groups (4–85 years) were susceptible to Adenovirus outbreak; however, interstingly the most vulnerable population group was categorized into those at age 15–40 [39]. It is not clear why the elderly are more resistant to severe disease acquisition; however, pre-existing neutralizing antibodies, and the weaker innate immunity which leads to less severe inflammatory conjunctivitis might be a possible explanation. However, AL-Mousawi’s study in Iraq was different as it showed the female gender as an important risk factor in groups [20]. Li et al. also revealed that the incidence was significantly higher among male students. They pointed out that the largest incidence of adenoviral keratoconjunctivitis infection was detected among 30–39-year-old (17.05%) people [21]. However, Shafiei et al. determined that the lowest incidence rate was in the age group of 10 years, which was different from our study [11]. This Iranian study showed that 94.4% of eye infection cases were caused by adenovirus, and there was no significant difference in terms of gender, age, and income level with the disease. These results are inconsistent with those of our study because some demographic characteristics, like gender and direct contact with an infected person in the family or friends, were risk factors for EKC [10]. More importantly, in the recent study some clinical finding was shown to be correlated with the Adenovirus infection such as membrane formation, conjunctival bleeding, tearing, and pain, as mentioned in Fig. 2, which has not been concluded in Shafiei’s study due to high percentage of positive Adenovirus samples.

There was a significant relationship between positive adenovirus tests and the history of infection in family or colleagues. In this regard, Li et al. reported that the main risk factor for infection was close contact with the patients or contaminated equipment [21].

The correlation between Adenovirus infection and clinical findings are poorly investigated in the clinic. The current study showed that conjunctival injection, eye discharge, conjunctival hemorrhage, tearing, follicular reaction, and foreign body sensation were the most common symptoms with more than 50% of cases involved. Das and Basu found redness (63.7%) and watery discharge (42.1%) as the commonest signs [22]. Epidemic keratoconjunctivitis is also the most severe form of conjunctivitis, and it presents with watery discharge, hyperemia, chemosis, and ipsilateral lymphadenopathy [23]. Preauricular lymphadenopathy or tenderness was documented in 1406 (7.3%) cases. Other studies pointed out that common manifestations of EKC were follicular hyperplasia, pseudomembrane formation, preauricular lymphadenopathy, corneal involvement, and blurred vision, which were different from the clinical findings in our study [21, 24]. Aoki et al. found that the presence of chemosis, subepithelial infiltration, and pre-auricular lymphadenopathy were more associated with EKC than with other ocular infections, such as bacterial or other viral agents involved in ocular infections [25]. They also reported pre-auricular lymphadenopathy and SEI in 23.5% and 43% of 68 cases, respectively, in adenoviral keratoconjunctivitis, which was due to several adenovirus types [25]. However, in the current study, there was no significant relationship between adenovirus detection and conjunctival injection, eye discharge, preauricular lymphadenopathy [25], and those symptoms which Lee et al. mentioned [26]. Also, there was no significant relationship between adenovirus genome detection and preauricular lymphadenopathy; which is inconsistent with the findings of Aoki et al. [25].

On the other hand, there was no significant relationship between clinical signs of the cornea and virus positive test. In a study, there was a correlation between clinical manifestations of EKC and viral infection [27]. The current results also showed that among clinical signs related to conjunctiva and eyelid, conjunctival bleeding and membrane formation had a significant relationship to positive PCR results. Aoki et al. and Hou et al. found different findings indicating that there was no significant relationship between PCR results and conjunctival bleeding and membrane formation signs [25, 28]. Also, pain and tearing had a significant relationship to PCR results, which was inconsistent with the results reported by Aoki et al. [25]. However, Lee et al. showed that there was a significant relationship between tearing and the existing adenovirus genome, as in our study [26].

Uemura et al. did not find a significant difference in clinical scores between human groups and several factors, such as days after onset, sex, HAdV DNA copy number on a logarithmic scale, and age [29]. Overall, we also did not find any related evidence regarding the association of other clinical manifestations with the presence of adenovirus in the molecular assessment. The inconsistency of the current study results with other reports on the relationship between clinical symptoms and positive molecular tests may be explained by some differences in sample size, the method of detection, or nonrandom sampling. Also, it was a single-center study from a referral center and needed more investigation in a larger population. Moreover, the absence of other adenovirus types as positive controls and relevant enterovirus genus during the test set-up were limitations.

The phylogenetic analysis revealed that all of the investigated sequences were classified as HAd-D8. The HAd8 was also detected as the major cause of many conjunctivitis outbreaks in different populations all over the world, such as Iran [11], Japan [30], Spain [31], Vietnam [32], Tunisia [33], China [34], and Turkey [7]. Sammons et al. showed that HAdV-8 was also the commonest genotype for epidemic conjunctivitis in neonatal patients [35]. Nguyen et al. also demonstrated that HAdV8 in Hanoi (VN2017) belonged to a subgroup of HAdV8 which is circulating in many parts of the world and their genomes are highly conserved [32].

Other studies have shown that the type of adenovirus might contribute to the severity of diseases as HAdv-8, HAdv-37, HAdv-53, HAdv-54, and HAdv-57 induce more inflammatory responses and illness than other types [36]. However, in our epidemics, the comparison between types was not applicable. Anyway, these results emphasized a critical need for the use of diagnostic methods to find more dangerous types and limit their spread [37].

This study had some limitations including a small sample size that came from a single center of the city, limited sequencing analysis, lack of a standard validated molecular test for primary screening, and lack of follow-up of cases.

In conclusion, the current study suggests that adenovirus is the most common cause of epidemic keratoconjunctivitis. Moreover, HAdV-D8 was the predominant circulating type in our area. In addition, demographic characteristics and clinical symptoms such as pain, tearing, membrane formation, lymphadenopathy around the ear, and conjunctival bleeding are associated with the presence of an adenoviral genome.

## Data Availability

The manuscript data would be available if requested by audiences.
